# In Vivo Reprogramming Using Yamanaka Factors in the CNS: A Scoping Review

**DOI:** 10.3390/cells13040343

**Published:** 2024-02-15

**Authors:** Han Eol Cho, Siwoo Lee, Jung Hwa Seo, Seong-Woong Kang, Won Ah Choi, Sung-Rae Cho

**Affiliations:** 1 Rehabilitation Institute of Neuromuscular Disease, Yonsei University College of Medicine, Seoul 06229, Republic of Korea; wtzephyr@yuhs.ac (H.E.C.); kswoong@yuhs.ac (S.-W.K.); 2 Department of Rehabilitation Medicine, Gangnam Severance Hospital, Seoul 06229, Republic of Korea; 3 Graduate Program of Biomedical Engineering, Yonsei University College of Medicine, Seoul 03722, Republic of Korea; tldndl98@gmail.com; 4 Department of Rehabilitation Medicine, Graduate School of Medical Science, Brain Korea 21 Project, Yonsei University College of Medicine, Seoul 03722, Republic of Korea; zugula@naver.com; 5 Research Institute of Rehabilitation Medicine, Yonsei University College of Medicine, Seoul 03722, Republic of Korea

**Keywords:** cellular reprogramming, central nervous system, Yamanaka factors, SRY-box transcription factor 2, octamer-binding transcription factor 4

## Abstract

Central nervous system diseases, particularly neurodegenerative disorders, pose significant challenges in medicine. These conditions, characterized by progressive neuronal loss, have remained largely incurable, exacting a heavy toll on individuals and society. In recent years, in vivo reprogramming using Yamanaka factors has emerged as a promising approach for central nervous system regeneration. This technique involves introducing transcription factors, such as Oct4, Sox2, Klf4, and c-Myc, into adult cells to induce their conversion into neurons. This review summarizes the current state of in vivo reprogramming research in the central nervous system, focusing on the use of Yamanaka factors. In vivo reprogramming using Yamanaka factors has shown promising results in several animal models of central nervous system diseases. Studies have demonstrated that this approach can promote the generation of new neurons, improve functional outcomes, and reduce scar formation. However, there are still several challenges that need to be addressed before this approach can be translated into clinical practice. These challenges include optimizing the efficiency of reprogramming, understanding the cell of origin for each transcription factor, and developing methods for reprogramming in non-subventricular zone areas. Further research is needed to overcome the remaining challenges, but this approach has the potential to revolutionize the way we treat central nervous system disorders.

## 1. Introduction

Central nervous system (CNS) diseases, particularly neurodegenerative disorders, stand as formidable challenges in the realm of medicine. These conditions, characterized by progressive neuronal loss, have remained largely incurable, exacting a heavy toll on individuals and society as a whole [1]. These debilitating conditions, characterized by the progressive loss of neurons, cast a long shadow over the lives of individuals and society as a whole. In the face of these challenges, the pursuit of effective therapies for CNS disorders has taken center stage in the realm of medical research.

In their landmark 2006 study, Takahashi and Yamanaka revolutionized regenerative medicine by demonstrating that a set of transcription factors—Oct4, Sox2, Klf4, and c-Myc—could reprogram differentiated fibroblasts into a pluripotent state similar to embryonic stem cells [2]. This pivotal discovery laid the foundation for induced pluripotent stem cell (iPSC) technology, challenging the long-held belief that cellular differentiation is irreversible. iPSC technology, both ethically sound and robust, allows differentiated cells to revert to a pluripotent state, subsequently differentiating into various cell types. This innovation has vast implications for tissue regeneration, disease modeling, and cell therapy by enabling the generation of patient-specific pluripotent cells. Especially for CNS disease, researchers can explore the genetic and sporadic aspects of conditions such as Alzheimer’s disease [3], amyotrophic lateral sclerosis [4], Huntington’s disease [5], and Parkinson’s disease [6] by deriving neurons from iPSCs.

Building on this concept, subsequent studies have explored the potential of direct reprogramming, a process that transforms one cell type directly into another without reverting to a pluripotent state. This method was initially demonstrated in 1987 with the discovery that the MyoD gene could reprogram fibroblasts into myoblasts [7]. Recent advancements have expanded the scope of direct transdifferentiation to include a variety of cells, such as transforming glial cells into functional neurons [8]. Direct reprogramming offers a faster and potentially safer alternative to iPSC technology, especially for generating specific cell types for therapeutic applications.

However, the majority of current neuroregeneration work, which predominantly relies on transplanting externally cultured cells, encounters significant challenges and uncertainties upon transplantation in vivo.

These challenges stem from the differences between experimental environments and the internal conditions of a living organism. For instance, cells differentiated in vitro do not always yield the same results when introduced into an in vivo context. Additionally, many stem cells face immune rejection or remain undifferentiated after transplantation [9].

To overcome these hurdles, the necessity for in vivo reprogramming has become increasingly apparent. In vivo reprogramming employs the internal cells for tissue repair, circumventing the complexities of cell culture and the significant risks of immune rejection linked to cell transplantation. This approach bypasses the need for external cell transplantation altogether, offering the exciting potential to convert endogenous non-neuronal cells within the CNS into functional neurons. This emerging technology represents a significant leap, bridging the gap between in vitro models and clinical applications, and holds immense promise for CNS repair and the development of novel therapeutic strategies. In the case of in vivo reprogramming, various transcription factors such as ASCL1 [10,11], NeuroD1 [11], and Neurogenin2 [12] are known to have the capacity to induce neurons within the CNS. On the other hand, there have been efforts to harness Yamanaka factors (Oct4, Sox2, Klf4, and c-Myc) to generate neural stem cells within the CNS, with the aim of regenerating the CNS. This potential to enhance cellular pluripotency through Yamanaka factors holds the exciting promise of achieving the appropriate transformation into various cell types essential for the CNS. In this review, we delve into the exciting world of CNS in vivo reprogramming, exploring the potential of Yamanaka factors to revolutionize the treatment of neurodegenerative disorders and transform the landscape of CNS medicine.

## 2. Materials and Methods

This review followed the Preferred Reporting Items for Systematic Reviews and Meta-Analyses extension for scoping reviews (PRISMA-ScR) statement [13]. The protocol of this scoping review was registered in the Center for Open Science (OSF) on 1 November 2023 (https://osf.io/pf7yh).

### 2.1. Literature Search

We conducted an extensive search of published scientific literature in databases including MEDLINE, EMBASE, Web of Science, and SCOPUS using the following search strategy until 1 November 2023: [((in vivo) OR (in situ)) AND (reprogramming) AND ((brain) OR (spinal cord)) AND ((sox2) or (oct4) or (c-myc) or (klf4))]. Only the titles, abstracts, or keywords were searched in SCOPUS. We applied no language restrictions in our search. To identify duplicate entries, we considered factors such as the author, publication year, article title, and the source’s volume, issue, and page numbers. Our search included studies of all types, including descriptive studies and case reports. Additionally, we manually reviewed the bibliographies of selected articles.

### 2.2. Study Selection and Eligibility Criteria

In vivo reprogramming studies that introduced Sox2, Oct4, c-Myc, and Klf4 directly into the CNS to regenerate cells were the primary focus of this research. To identify relevant studies, a comprehensive two-stage screening process was implemented.

#### 2.2.1. Stage 1: Title and Abstract Screening

Two independent reviewers initially screened the titles and abstracts of all retrieved articles. Any discrepancies in assessments were resolved by a third reviewer. Articles deemed relevant based on initial screening proceeded to full-text evaluation.

#### 2.2.2. Stage 2: Full-Text Evaluation

During the full-text evaluation phase, the reviewers critically assessed the eligibility of each study against a set of pre-established inclusion and exclusion criteria:

Inclusion Criteria:

1. Articles reporting the use of one or more of the reprogramming factors, Sox2, Oct4, c-Myc, and Klf4, for inducing in vivo reprogramming in the CNS (brain and spinal cord).
2. Peer-reviewed articles written in English.

Exclusion Criteria:

1. Articles focused on in vitro exams.
2. Articles involving the transplantation of cells induced through in vitro reprogramming.
3. Articles related to reprogramming other than CNS lesions.
4. Non-original articles (such as reviews), editorials, letters from editors, book chapters, unpublished or non-peer-reviewed studies, abstracts, and PhD theses.
5. Articles for which the full text was not accessible.

Any discrepancies between reviewers during full-text evaluation were resolved through discussion and consensus. This rigorous two-stage screening process ensured the selection of high-quality, relevant studies that aligned with the research objectives.

### 2.3. Data Extraction

The reviewers conducted an in-depth analysis of the full-text articles, extracting key information from relevant studies. These details included the first author, publication year, title, journal, the transcription factors used, animal models, the method of transcription factor delivery, the cell of origin, the target-induced fate, a description of the main findings, and the study’s conclusions.

## 3. Results

### 3.1. Study Selection

**Figure 1:**
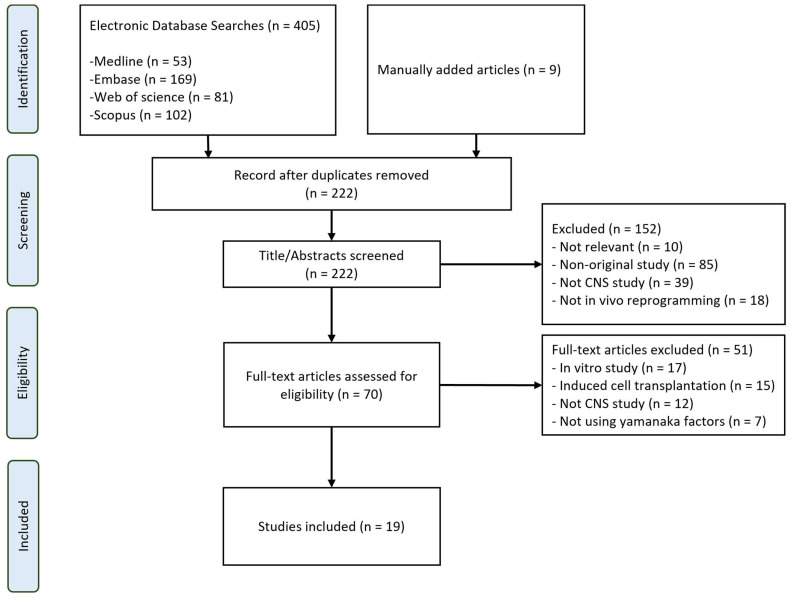
Flowchart of search strategy used in this study.

The process of reviewing articles and extracting data followed the structure outlined in Figure 1, as represented in the PRISMA-ScR flow diagram. Initially, a total of 405 articles were identified across the four selected databases using the designated search strategy. In addition, nine articles were obtained and included through manual searching. Excluding duplicates, there were initially 222 papers. After a review of titles and abstracts, 152 articles were further removed. Subsequent full-text screening led to the exclusion of 51 more articles, leaving a total of 19 articles. 

### 3.2. In Vivo Reprogramming Study Using All Four Yamanaka Factors

In a total of four studies, Oct4, Klf4, Sox2 and c-Myc(OKSM) were employed simultaneously. These included papers are summarized in Table 1.

#### 3.2.1. Healthy Animal Models

In the 2020 study conducted by Rodriguez et al. [14], researchers used reprogrammable mice to investigate whether the expression of Yamanaka factors is associated with the induction of aging markers in the dentate gyrus. Observing that the continuous expression of OKSM led to an increase in premature death, they tested for a cyclic protocol (active for 3 days, followed by a 4-day rest, over 15 cycles) from 6 months to 10 months of age. When OKSM factors were cyclically expressed, there was an observed increase in migrating cells containing the neurogenic markers doublecortin (DCX, marker for immature neurons) and calretinin. Furthermore, H3K9me3, typically decreasing in the dentate gyrus with age, showed a smaller reduction, alongside an increase in the GluN2b subunit within NMDA receptors. Notably, after five days of treatment, the OKSM group displayed a significant improvement in memory index, a change found to be proportional to the duration of exposure.

#### 3.2.2. Disease or Injured Animal Models

In Wi et al.’s 2016 study [16], high concentrations of OKSM were directly injected into the lateral ventricles of HBI mice. The treatment group exhibited increased proliferative cells, with these proliferative cells showing a 3.1-fold increase in βIII-tubulin (early neuronal marker)-positive cells and a 6.2-fold increase in Neun (mature neuronal maker)-positive cells. Additionally, there was a 4.3-fold increase in Nestin (neural progenitor marker)-positive cells and a 2.9-fold increase in GFAP (astrocyte marker)-positive cells observed in the subventricular zone. Moreover, hippocampal synaptic plasticity was enhanced, and a functional improvement was noted in treated mice, which included improvements in long-term memory, anxiety, and other aspects of functionality.

In the same group’s other 2016 study, they conducted experiments using a cerebral ischemia model in mice [17]. They utilized reprogrammable mice in which the four pluripotency factors OKSM were expressed in the presence of doxycycline. They precisely positioned an infusion cannula in the lateral ventricle using stereotaxic methods. The effects of different concentrations were then assessed by infusing either low (1 μg/mL; DOX-L) or high (100 μg/mL; DOX-H) concentrations of doxycycline or PBS (as a solvent control) into the lateral ventricle via a micro-osmotic pump.

The high expression of OKSM led to increased expression of neural progenitor cells in the subventricular zone. Furthermore, it promoted the proliferation of astrocytes in the subventricular zone and striatum, but it did not increase glial scar formation. There was also an increase in neovascularization in the striatum, and in the high-expression group, there was an increase in Neun+ cells and the expression of PSD95 (a synaptic marker). Finally, motor function also improved as a result of these findings.

In the 2016 study by Gao et al., they focused on in vivo reprogramming in the brain cortex of traumatic brain injury mice [15]. Using retroviruses, they induced the expression of OKSM in reactive glial cells. This resulted in the expansion of cell clusters, which subsequently transformed into NanoG (a marker for embryonic stem cells)- or SSEA4 (cell surface marker expressed in the embryonic stem cells)-positive embryonic stem cell-like cells and further differentiated into various cell types. At 4 weeks, they observed the formation of neural tube-like structures, along with the presence of Nestin+ neural stem cells and DCX+ cells. By 6 weeks, Neun+ and Map2 (mature neuron markers)-positive mature neurons were detected, and these neurons exhibited electrophysiological activity, indicating that they were functional neurons. The reprogramming also led to the conversion into astrocytes and oligodendrocytes but not into microglia.

#### 3.2.3. Safety Concerns Associated with the Use of All Four Yamanaka Factors

Interestingly, when all OKSM factors were used, potential side effects were easily observed. In Gao’s study, teratomas emerged after 8 weeks [15], while in Rodriguez’s research, the continuous expression of OKSM led to premature death [14].

### 3.3. In Vivo Reprogramming Study Using Oct4

A total of five studies utilized Oct4 as the sole transcription factor (Table 2).

**Table 2:** In vivo reprogramming study using Oct4.

Reprogramming Factors	Expression Location	Animal Model /Lesion Model	Animal Age (Time of Reprogramming *)	Delivery Methods		Target Cell (Markers)	Functional Outcome	References
Oct4	Dentate gyrus	C57BL/6 male mice	8 weeks old	Lentivirus	Stereotactic injection	-	Behavioral test (open field test, elevated plus maze, Y-maze test, contextual fear conditioning paradigm)	[18]
Oct4 + VPA	Lateral ventricle	C57BL/6 mice	8~9 weeks old	Lentivirus	Stereotactic injection	Neural stem cell (Pax6, Sox1) Pluripotency marker (Oct4, Nanog, c-Myc, Klf4 and Sox2)	-	[19]
Oct4 + VPA	Lateral ventricle	C57BL/6 mice	8~9 weeks old	Lentivirus	Stereotactic injection	Neural progenitor and pluripotency markers (Oct4, Nanog, Klf4, c-Myc, Pax6 and Sox1, SSEA1,Nanog)		[20]
Oct4 + VPA	Lateral ventricle	C57BL/6 mice	not mentioned (1 week before inducing demyelination)	Lentivirus	Stereotactic injection	Myelinating oligodendrocytes	Visual evoked potential	[21]
		Optic chiasm demyelination by 1% lysolecithin						
Oct4	Lateral ventricle	R6/2 mice	4 weeks old	Adenovirus	Stereotactic injection	Neuron (NeuN (cortex) GAD67, Darpp32 (striatum))	Behavioral test (Rotarod test, Grip strength test)	[22]
		Huntington’s disease model						

VPA, valproic acid. * if applicable, any additional specific time points.

**Table 3:** In vivo reprogramming study using Sox2.

Reprogramming Factors	Expression Location	Animal Model /Lesion Model	Animal Age (Time of Reprogramming *)	Delivery Methods		Target Cell (Markers)	Functional Outcome	References
Sox-induced in vivo brain reprogramming								
Sox2 + BNDF/noggin or VPA	Striatum	C57BL/6J and ICR mice hGFAP–Cre, mGfap–Cre line 77.6, Nes–CreERTM, NG2–Cre, PrP–CreERT, Rosa–YFP, Rosa–tdTomato (Ai14)	Between 6 weeks and 24 months	Lentivirus	Stereotactic injection	Neuron (NeuN)	Functional electrophysiology	[23]
Sox2	Striatum	C57BL/6 and ICR mice Tlxflox/flox mice transgenic pGFAP-Cre mice	Not mentioned	Lentivirus	Stereotactic injection	Neuron (DCX)		[24]
Sox2/VPA	Striatum	Cst3-CreERT2, Nes-CreERTM, Ascl1-CreERT2, Ascl1neoflox/neoflox, Rosa-YFP, and Rosa-tdTomato	2–6 months of age	Lentivirus	Stereotactic injection	Neuron (NeuN, Calretrin)		[25]
Sox2 + Nurr1 + Lmx1a + Foxa2 + VPA	Striatum	C57BL/6J mice mGfap-Cre line 77.6, PrP-CreERT, Pdgfra-CreERT, Dat-Cre, and Rosa-tdTomato (Ai14)	6 weeks to 24 months	Lentivirus	Stereotactic injection	Dopaminergic neuron	Electrophysiological Properties and firing patterns, network connectivity	[26]
Sox2 ± ASCL1	Cerebral cortex	C57BL/6J mice Sox10-iCreERT2/GFP or GLASTCreERT2/GFP mice	8–10 weeks old (3 days after stab wound injury)	Retrovirus Lentivirus	Stereotactic injection	Neuron (DCX, NeuN)		[27]
		Stab Wound Lesion						
Sox2	Corpus callosum(left)	C57BL/6J mice	12 weeks old	Lentivirus	Stereotactic injection	Oligodendrocyte precursor cells (PDGFRα+) oligodendrocytes		[28]
		Demyelination induced by 0.2% Cuprizone in diet chow						
Sox-induced in vivo spinal cord reprogramming								
Sox2/VPA	Spinal cord	C57BL/6J and the immunodeficient NSG mice	2–3 months of age ((immediately after hemisection)	Lentivirus	Manual injection (into the spinal cord parenchyma at each of the two locations 3 mm apart at the T8)	Neuron (NeuN, MAP2), Synapse-forming GABAergic interneurons		[29]
		hemisection at the T8 level						
Sox2	Spinal cord	C57BL/6J mice Ptenflox, p53flox, p21 KO, mGfap-Cre line 77.6, Thy1-STOP-YFP, Rosa-tdT	8 weeks and older	Lentivirus	Manual injection (into the spinal parenchyma at each of the two locations 2 mm apart at the T8 level)	Neuron (NeuN, MAP2)		[30]
		contusion injury at the T7–9 level						
Sox2	Spinal cord	ICR mice	8 weeks old (1 week after SCI)	Adenovirus	Manual injection (1.0 mm caudal and rostral to the lesion site)	Neuron (Nissl and βIII-tubulin)	Behavioral test (BMS score, Running wheel test, Swimming test, Inclined plate test, Mechanical allodynia test)	[31]
		Completely compression for 5 s at T10 level						
Sox2	Spinal cord	C57BL/6J mice Rosa-YFP, Rosa-tdT, Sox2f/f, Pdgfra-CreER™, Ascl1-CreERT2, Nes-CreERT2, Foxj1-CreERT2, Rosa-TVAg mouse line	2 months of age and older	Lentivirus	Manual injection (0.5 mm rostral and caudal to the incision, bilaterally)	Neuron (NeuN, VGLUT2, GAD6, VGAT)	Behavioral test (Grid-walking test)	[32]
		dorsal hemisection at the C5 level						

* if applicable, any additional specific time points.

#### 3.3.1. Healthy Animal Models

Sim S. et al. overexpressed Oct4 to investigate changes in the dentate gyrus and behavioral alterations, but the results did not yield significant findings [18].

Javan M.’s team conducted several studies on Oct4-driven reprogramming [19,20,21]. In their 2015 paper published in *Cell Journal* [19] and *Life Science* [20], they investigated the effectiveness of a combination therapy involving Oct4 and small molecules. Valproic acid (VPA), BIX-01294, Bay K8644, and RG-108 are chemicals that have been identified as influential in enhancing reprogramming efficiency or substituting certain reprogramming factors in in vitro research [33,34]. Upon administering exogenous Oct4 to the right cerebral ventricle, they observed an increase in markers such as NanoG, Klf4, c-Myc, Pax6, and Sox1, which became significantly enhanced when combined with VPA [19,20]. However, the co-administration of BIX-01294, Bay K8644, and RG-108 did not yield a synergistic effect, and when added to Oct4 + VPA, these compounds even reduced the expression of the earlier markers [19].

Interestingly, the simultaneous administration of VPA and Oct4 from 7 days before exogenous Oct4 significantly increased markers of neural stem cells such as Pax6 and Sox1, along with pluripotent indicators like endogenous Oct4, Nanog, Klf4, and c-Myc. This combinational treatment of VPA and Oct4 led to the reprogramming of endogenous somatic cells in the brain rather than inducing the proliferation of endogenous neural stem cells [19,20]. Moreover, through immunohistochemical analysis, it was confirmed that astrocytes were the main type of transfected cells, leading to the inference that astrocytes were the cell of origin.

#### 3.3.2. Disease or Injured Animal Models

One study showed the in vivo reprogramming effects of Oct4 in mice with optic chiasm demyelination. In this study, mice received oral administration of VPA for a week, followed by the injection of lentiviral particles capable of inducing Oct4 expression into the lateral ventricle. Subsequently, one week post-Oct4 induction, LPC was administered into the optic chiasm to induce demyelination. At 7 days post-injury, the group that received pre-VPA + Oct4 treatment showed a significantly reduced extent of demyelination. Furthermore, the expression of Oct4 enhanced myelination by converting transduced cells into myelinating oligodendrocytes. When assessing the recovery of the optic chiasm through visual evoked potentials, it was confirmed that the pre-VPA + Oct4 group exhibited the restoration of visual evoked potentials [21].

In a 2021 study by Yu et al., the reprogramming effects of Oct4 were investigated using R6/2 mice, a Huntington’s disease model [22]. Two weeks after Oct4 injection, an increase in Nestin+ cells, a marker of neural stem cells, was observed. Furthermore, there was an increase in NG2+ cells, a marker of oligodendrocyte precursor cells. By the 13th week, the AAV9-Oct4 group exhibited a substantial upregulation of markers related to oligodendrocyte precursor cells, including NG2, Olig2, PDGFRα, Wnt3, MYRF, and GDNF. When assessed using transmission electron microscopy and magnetic resonance imaging, a reduction in myelination defects was observed. Additionally, increased expression of markers associated with neurons (b3 tubulin, Neun) and GABAergic neurons (GAD67, and DARPP32) was confirmed. The study also involved a serial assessment of behavioral performance in mice. Notably, the Oct4 group showed significantly improved motor function between weeks 8 and 13. In conclusion, this research demonstrated that Oct4 overexpression in a Huntington’s disease mouse model increased neural stem cells in the subventricular zone, expanded the oligodendrocyte lineage, promoted GABAergic neuron formation, reduced myelin defects, and positively impacted functional outcomes.

#### 3.3.3. Safety Concerns Associated with the Use of Oct4

In the entire study, no specific safety issues were reported. One study disclosed that no teratoma formation was observed even 100 days post-infection, thereby confirming the safety of Oct4 injection [20].

### 3.4. In Vivo Reprogramming Study Using Sox2 in the Brain

There were six in vivo reprogramming studies about the effects of Sox2 alone in the brain. According to studies, Sox2 alone can induce the transformation of non-neuronal cells into DCX+ neurons (Table 3).

#### 3.4.1. Healthy Animal Models

Zhang, C. L. and colleagues conducted research on reprogramming in the brain. In their 2013 study, they confirmed that DCX+ induced adult neuroblasts could be induced in the striatum using Sox2 alone [23]. In their 2015 study, they demonstrated that the use of Sox2 transformed striatal astrocytes into ASCL1+ neural progenitors, which subsequently progressed into DCX+ induced adult neuroblasts [25]. These induced adult neuroblasts showed proliferative activity after reprogramming [23]. Furthermore, they demonstrated that Sox2 alone was insufficient for the formation of mature neurons, particularly Neun+ neurons [23,25]. However, when combined with additional factors such as the neurotrophin Bdnf and the bone morphogenetic protein inhibitor Noggin [23] or with the histone deacetylase inhibitor VPA [25], these cells could overcome the apparent barrier preventing further neuronal maturation in the brain. These induced neurons were identified as calretinin+ and Neun+ neurons, and they were detected for up to 36 weeks. Moreover, these induced neurons displayed electrophysiological functionality and integrated into local circuits, allowing them to receive inputs from presynaptic neurons [22]. Regarding the cell of origin, Zhang C. L.’s group suggested astrocytes as the source. However, Heinrich’s study proposed that NG2 glial cells were the origin of induced neurons.

There have been studies exploring the factors involved in Sox2-induced in vivo reprogramming. In their study, Islam, M. M. and colleagues provided evidence that Tlx expression in astrocytes significantly reduced the detection of Sox2-induced DCX+ cells in the adult striatum, which implies that Sox2-regulated Tlx expression is required for the in vivo reprogramming process [24]. Niu et al. demonstrated that the deletion of ASCL1 significantly reduced the number of DCX+ cells induced by Sox2 reprogramming [25]. While ASCL1 plays a critical role in Sox2-driven reprogramming, it is not sufficient on its own to trigger a complete cell fate switch.

Niu et al. used Sox2 in a different reprogramming pathway while aiming to generate dopaminergic neuron-like cells [26]. When they added FOXA2, LMX1A, or NURR along with VPA to Sox2, they observed the expression of TH+ cells. Notably, these induced dopaminergic neuron-like cells did not originate from NG2 glia, astrocytes, resident glial cells, or neurogenic neural progenitors in the subventricular zone. Instead, they were derived from endogenous (local) striatal neurons. The induced dopaminergic neuron-like cells expressed DARPP32 and CTIP2 and exhibited electrophysiological properties and firing patterns similar to dopaminergic neurons. They were also functionally connected to other neurons, indicating their similarity to dopaminergic neurons in terms of functional properties.

#### 3.4.2. Disease or Injured Animal Models

Heinrich’s 2014 study showed that Sox2-induced immature neurons were formed in a stab wound injury of the cortex [27] from NG 2 glial cells. These induced neurons also exhibited immature neuronal activity as evidenced by electrophysiological analysis.

In the study conducted by Farhangi et al. in 2019, they demonstrated that Sox2 could facilitate the conversion of astrocytes into oligodendrocyte precursor cells, ultimately leading to myelinating cells (PDGFRa+) in a multiple sclerosis model [28].

#### 3.4.3. Safety Concerns Associated with the Use of Sox2 in the Brain

Regarding tumor formation, two studies conducted follow-ups for up to 50 weeks after Sox2 injection but neither observed tumor formation [23,25].

### 3.5. In Vivo Reprogramming Study Using Sox2 in Spinal Cord Injury Models

There was a total of four studies that conducted in vivo Sox2-induced reprogramming in the spinal cord(Table 3). All studies targeted animal models with spinal cord injuries. Sox2 alone can also robustly reprogram endogenous NG2 glia toward DCX+ cells in the adult mouse spinal cord [29,31,32]. This could be observed in older mice as well.

The cell of origin varied among the studies. Two studies showed that astrocytes were identified as the cell of origin [29,31]. Su et al. demonstrated that astrocytes could be reprogrammed by Sox2 into neural progenitors, which then proceeded through a proliferative DCX+ neuroblast stage to become DCX+ neurons [29]. When VPA was used, they were further converted into GABAergic neurons. Yang et al. showed efficient reprogramming of scar-forming astrocytes into neurons, with a conversion rate of 22.1% [31].

Tai et al. proposed that NG2 glial cells, rather than astrocytes, were the cells of origin [32]. They observed that NG2 glia, when subjected to Sox2 induction, went through proliferative progenitor and neuroblast states, eventually differentiating into various neuronal subtypes. These induced neurons expressed pre-synaptic (SYN1+), excitatory (VGLUT2+), inhibitory (GAD6+, VGAT+), and glycinergic markers (GLYT2). Additionally, these NG2 glia-derived neurons were capable of forming monosynaptic connections with propriospinal neurons, as well as neurons located in the brainstem and dorsal root ganglia (DRGs), which are involved in the ascending and descending pathways.

Nevertheless, using Sox2 alone for the conversion into mature neurons proved to be relatively ineffective. It became evident that DCX+ cells could efficiently mature into functional neurons when supplemented with additional factors such as VPA [29], BDNF-Nog, or p75-2 (a mutant variant of the neurotrophic factor NT with reduced affinity for p75NTR) [32]. This suggests that, similar to the brain, Sox2 alone can initiate the transformation into immature neurons in the spinal cord, but supplementary factors are imperative to facilitate their maturation into fully mature neurons.

The use of Sox2 facilitated recovery in spinal cord injury. Sox2 usage in spinal cord injury showed reduced scar formation and improved functional levels [31,32]. While one study showed that sox2 treatment alone did not lead to significant functional recovery, subsequent rehabilitation significantly improved functional outcomes, suggesting the potential of combined rehabilitation and Sox2 treatment [31].

One study showed that the p53-p21 pathway played a crucial role in the generation of induced adult neuroblasts from astrocytes in spinal cord Sox2-mediated reprogramming. Silencing the p53-p21 pathway significantly increased the formation of DCX+ cells, but maturation into Neun+ cells did not occur [30].

## 4. Discussion

This scoping review has provided a comprehensive overview of the promising potential of in vivo reprogramming for the CNS using Yamanaka factors.

CNS diseases often result in devastating consequences and, unfortunately, the CNS lacks inherent regenerative capacity. The induction of differentiated cells into pluripotent stem cells presents a novel approach for treating CNS diseases, as these cells possess the unique ability to differentiate into various cell types including neurons. Reprogramming is typically achieved by introducing specific transcription factors (such as Oct4, Sox2, Klf4, and c-Myc) into adult cells, resetting the cells to an embryonic-like state [2]. A variety of in vitro studies have demonstrated the potential for various cell types to be converted into iPSCs, with a specific focus on their differentiation into neurons [3]. The use of cell transplantation with these induced cells has garnered significant interest as a potential treatment approach for CNS diseases. However, concerns regarding the ethical implications [35] and the risk of immune rejection associated with human fetal tissues have been addressed through the development of iPSCs derived from a patient’s own somatic cells [36]. Furthermore, persistent concerns about teratoma formation due to iPSCs remain a consideration [37].

Using Yamanaka factors for in vivo reprogramming offers significant advantages. First, this approach involves patient-specific cells, eliminating concerns about immune rejection. Moreover, it reduces the risk of teratoma formation. While the use of all four factors (OKSM) can lead to adverse effects, two of the Yamanaka factors, c-Myc and Klf4, are known oncogenic factors and may promote tumor development [38,39]. In cases where only Sox2 and Oct4 are used for reprogramming, no tumor formation was reported during extended monitoring periods. This suggests that in vivo reprogramming using Sox2 and Oct4 is considered a safer approach.

There have been efforts in the CNS to regenerate neurons through in vivo reprogramming using transcription factors other than Yamanaka factors, such as NeuroD1 [40,41,42,43] and Neurogenin 2 [44,45,46]. These transcription factors are known to lead to the direct transformation of astrocytes into neurons. Using these factors has shown relatively rapid neuronal expression. In one study using NeuroD1, neuronal markers began appearing as early as 11 days [43]. In another study using NeuronD1, mature neuron markers like Neun+ were detected after one week [40]. With Neurogenin 2, the differentiation of neural progenitors mediated by Neurogenin 2 is swift, resulting in detectable neurons as early as 3 days post-injection, although most induced neurons cannot survive beyond 56 days, even with additional BDNF [46].

In contrast, research on in vivo reprogramming using Yamanaka factors suggests a comparatively slower process. Although the timeframe for detecting neurons varies across studies, it generally takes several weeks. Su et al. showed that DCX+ neurons were observed between 4 to 8 weeks, while Neun+ neurons were detectable around 8 weeks [29]. Similarly, Gao et al. found DCX+ cells around 4 weeks and Neun+ cells at 6 weeks [15]. Another showed progenitors appearing at 3 weeks and DCX+ cells at 4 weeks [32]. We supposed that these differences might stem from varying stages of transformation. The processes involving Sox2 or Oct4 potentially facilitate the proliferation and differentiation of neural progenitor cells within the CNS into neurons. This approach, which involves the expansion and differentiation of neural progenitor cells rather than directly converting glial cells into neurons, could result in a slower process. Niu et al.’s research supports this approach; they showed that induced adult neuroblasts were detectable from 1 to 3 weeks, peaked at 7 weeks, and persisted until 14 weeks after using Sox2 [23]. Moreover, Sox2-induced cells showed sustained presence; one study detected Sox2-induced mature neurons from astrocytes that were still observable at 210 days post-injection when the mice were treated with VPA [29]. Further comparative studies through clearer time-series analyses will be necessary to delineate the differences among these transcription factors.

Neural stem cells and progenitors in the adult brain, particularly in the subventricular zone, hippocampal dentate gyrus, and brain parenchyma, contribute to CNS repair. However, their effectiveness is limited in extensive and chronic lesions [47]. Our review reveals that reprogramming works well in the subventricular zone but is less effective in the dentate gyrus [14,18]. Some cortex studies showed reprogramming, but it required prior lesions [27]. Future research should explore reprogramming mechanisms, extend beyond the subventricular zone, and find methods for reprogramming in non-subventricular-zone areas.

The key factor in reprogramming is its ability to produce clinically functional neurons and achieve functional improvements. Several studies have utilized Yamanaka factors to generate mature neurons, confirming their electrophysiological functionality and their formation of functional synapses within the local neural network. Significantly, various studies have expanded these findings to behavioral tests, demonstrating tangible improvements in models of neurological disorders. This direct correlation between cellular reprogramming and behavioral outcomes emphasizes the potential of these techniques not only in neuronal network reconstruction and tissue repair but also in enhancing functional outcomes in disease models. These discoveries underscore the transformative potential of in vivo reprogramming in regenerative medicine, paving the way for effective treatments for various neurodegenerative diseases.

Although most SOX2 research identifies astrocytes as the cell of origin, there is some evidence suggesting NG2 cells. Conversely, Oct4 studies have not delved as deeply into this aspect. Gao’s work [15] postulates the involvement of reactive glial cells, while Dehghan [20] points to astrocytes. Tai et al. [32] presented findings in their research that diverge from previous studies by identifying NG2 cells as the cell of origin. They suggest that the levels of endogenous SOX2 after spinal cord injury may not surpass the threshold required for effective neurogenic reprogramming of reactive astrocytes. This indicates that different cell types may necessitate varying SOX2 levels, or other factors in astrocytes might maintain their fate in a more stable state. Moreover, glial cell responses to injuries might differ between regions, as illustrated by the lack of DCX expression in the injured adult mouse brain cortex, which contains a high proportion of reactive glial cells.

Understanding the precise lineage in reprogramming is essential. The study by Wang et al. raised concerns about the reliability of viral targeting strategies in the lineage tracing of new neurons using AAV, leading to obscurities in the interpretation of many reports. To ascertain the true origin of viral-reporter-labeled neurons, more reliable methods like lineage tracing are necessary.

Future investigations should prioritize precise determination of the cell of origin, possibly examining the influence of specific transcription factor concentrations. Furthermore, studies should explore strategies for optimizing transcription to enhance the efficiency of reprogramming.

Both Sox2 and Oct4 displayed significantly increased reprogramming efficiency when used in conjunction with VPA, especially in promoting the transformation of cells into mature neurons. VPA, a histone deacetylase inhibitor known for its roles in triggering BDNF expression and encouraging neural differentiation [48], played a vital role in enhancing this process. The heightened efficacy of VPA may be partly due to the greater activity of histone deacetylases in mouse cells. It is also worth noting that maturation can be further enhanced using BDNF-Noggin or p75-2. Additional research in this area is essential to deepen our understanding of these underlying mechanisms.

### Challenges and Future Directions

Despite harboring immense potential, in vivo reprogramming currently faces several formidable challenges. Firstly, the conversion rate and survival of in vivo reprogrammed cells are pivotal. It is crucial to not only generate an adequate number of functional neurons of various subtypes but also ensure these neurons establish proper synapses with existing cells, sustain longevity, and effectively contribute to the restoration of brain and spinal cord functionalities. While certain studies have demonstrated behavioral improvements, additional research is imperative to amplify these outcomes.

Secondly, the capability to induce a broader array of neuron subtypes tailored to specific needs remains a significant hurdle. Some strides have been made in regenerating glutamatergic or GABAergic neurons, and even dopaminergic neurons in certain instances. However, there is a pressing need to devise and refine methods for producing other neuron types such as cholinergic, serotonergic, and norepinephrinergic neurons with greater efficiency.

Thirdly, addressing spatial challenges, particularly within the brain, is crucial. The bulk of research to date has concentrated on regions like the striatum and lateral ventricle, which are reputed for harboring intrinsic stem cells. There is a dire need for studies exploring how newly formed neurons can accurately project their axons to the intended targets. In the spinal cord context, it is essential to integrate regenerated cells with pre-existing neurons to enhance connections between upper and lower neural circuits.

Finally, the development of safer delivery mechanisms is essential for the clinical application of reprogramming strategy technologies. Recent studies, both in vitro and in vivo, have demonstrated the potential of viral vectors for therapeutic use. However, concerns about viruses, including immunogenicity and mutagenesis risks such as oncogenesis, remain. Consequently, current research into in vivo reprogramming using non-viral vectors is vital. These include methods such as polymers, liposomes, and viral-like particles. We are hopeful for the advancement of technologies that can harness the power of in vivo cell regeneration through safe methods, potentially bringing transformative changes to healthcare.

## 5. Conclusions

In conclusion, our review highlights the promising potential of treating CNS diseases through cellular regeneration via in vivo reprogramming with Yamanaka factors, especially Sox2 and Oct4. This approach may offer advantages in clinical applications as it eliminates the need for exogenous cells and transplantation. Further research is essential to explore methods for increasing conversion into neurons, investigate cell origins, and understand the unique in vivo reprogramming processes within the CNS environment, distinct from in vitro conditions.

## Figures and Tables

**Table 1:** In vivo reprogramming using all four Yamanaka factors.

Reprogramming Factors	Expression Location	Animal Model/Lesion Model	Animal Age (Time of Reprogramming *)	Delivery Methods		Target Cell (Markers)	Functional Outcome	References
OKSM	Dentate gyrus	Reprogrammable i4F-B mice (with a C57BL/6 genetic background)	6 months old (6 to 10 months of age)	Doxycycline-inducible	3 days on doxycycline, then 4 days off for 15 weeks	Levels of migrating cells	Object Recognition Test	[14]
OKSM	Cerebral cortex	C57BL/6 mice	12 weeks old (3 days after TBI)	Retrovirus	Stereotactic injection	Neuron (NeuN, Map2)	Functional electrophysiology	[15]
		Controlled cortical impact TBI)						
OKSM	Lateral ventricle	ICR mice	6 weeks old	Adenovirus	Stereotactic injection	Neuron (NeuN)	Behavioral test (Passive Avoidance Task, open field test)	[16]
		Chronic Hypoxic–Ischemic Brain Injury model (unilaterally carotid artery ligation at 1 week of age)						
OKSM	Lateral ventricle	Reprogrammable i4F-B mice (with a C57BL/6 genetic background)	8–16 weeks (immediately after cerebral ischemia)	Doxycycline-inducible	Infused doxycycline into the lateral ventricle for 7 days using an osmotic pump	Neuron (NeuN)	Behavioral test (Rotarod test, ladder walking test)	[17]
		Cerebral ischemia model (bilateral common carotid artery occlusion for 20 min)						

TBI, traumatic brain injury; i4F-B, doxycycline-inducible polycistronic cassette encoding the four murine factors Oct4, Sox2, Klf4, and c-Myc. * if applicable, any additional specific time points.
